# Structure-Function Studies of Glucose Oxidase in the Presence of Carbon Nanotubes and Bio-Graphene for the Development of Electrochemical Glucose Biosensors

**DOI:** 10.3390/nano14010085

**Published:** 2023-12-28

**Authors:** Christina Alatzoglou, Eleni I. Tzianni, Michaela Patila, Maria G. Trachioti, Mamas I. Prodromidis, Haralambos Stamatis

**Affiliations:** 1 Biotechnology Laboratory, Department of Biological Applications and Technologies, University of Ioannina, 45110 Ioannina, Greece; ch.alatzoglou@uoi.gr (C.A.); mpatila@uoi.gr (M.P.); 2 Laboratory of Analytical Chemistry, University of Ioannina, 45110 Ioannina, Greece; el.tzianni@gmail.com (E.I.T.); trachioti93@gmail.com (M.G.T.)

**Keywords:** glucose oxidase, multi-walled carbon nanotubes, bio-graphene, structure-function studies, electrochemical glucose biosensor

## Abstract

In this work, we investigated the effect of multi-walled carbon nanotubes (MWCNTs) and bio-graphene (bG) on the structure and activity of glucose oxidase (GOx), as well as on the performance of the respective electrochemical glucose biosensors. Various spectroscopic techniques were applied to evaluate conformational changes in GOx molecules induced by the presence of MWCNTs and bG. The results showed that MWCNTs induced changes in the flavin adenine dinucleotide (FAD) prosthetic group of GOx, and the tryptophan residues were exposed to a more hydrophobic environment. Moreover, MWCNTs caused protein unfolding and conversion of α-helix to *β*-sheet structure, whereas bG did not affect the secondary and tertiary structure of GOx. The effect of the structural changes was mirrored by a decrease in the activity of GOx (7%) in the presence of MWCNTs, whereas the enzyme preserved its activity in the presence of bG. The beneficial properties of bG over MWCNTs on GOx activity were further supported by electrochemical data at two glucose biosensors based on GOx entrapped in chitosan gel in the presence of bG or MWCNTs. bG-based biosensors exhibited a 1.33-fold increased sensitivity and improved reproducibility for determining glucose over the sweat-relevant concentration range of glucose.

## 1. Introduction

The need for innovative bioanalytical platforms, such as electrochemical enzyme-based glucose biosensors, is urgent in the field of medicine for the effective and rapid determination of glucose levels in various biofluids [1,2]. These devices present several advantages such as high sensitivity, fast response, and low cost of production. Electrochemical glucose biosensors can be fabricated by homogeneous or heterogeneous methods, depending on whether a nanomaterial interferes between the electrode and the enzyme that acts as the signal molecule [3,4]. The development of homogenous biosensors is simple, inexpensive, and exploits the adsorptive capacity and binding ability of the reactants towards the electrode; these biosensors surpass steric hindrance and improve enzymatic efficiency. On the contrary, the fabrication of heterogeneous biosensors relies on the modification of the electrode with nanomaterials and the subsequent attachment of the enzymes; this way, nanomaterials serve as an effective electron transport between the signal molecule and the electrode, which endows these biosensors with high sensitivity, stability, and multiplexed detection [3,5]. The materials involved in various biosensing architectures aim to provide a suitable microenvironment for the immobilized enzymes to retain their activity, as well as an increased electron transfer between the redox center of the immobilized enzymes or the electroactive species involved in the analysis (mediators, enzyme reaction products, etc.) and the electrode surface [1].

**Figure 1:**
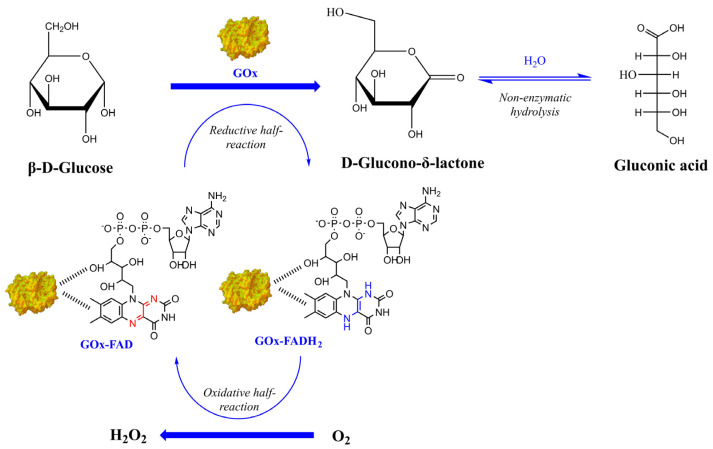
Representation of the glucose oxidation reaction catalyzed by GOx (adapted from [12]).

Glucose biosensors often involve the immobilization of glucose-1-dehydrogenase (GDH) or glucose oxidase (GOx) on electrodes modified with different nanomaterials; however, the use of GOx is mostly preferred, as this enzyme exhibits higher affinity and sensitivity towards glucose and remains stable at wider ranges of temperature and pH [6]. GOx (EC1.1.3.4) is an oxidoreductase composed of two identical subunits; each monomer non-covalently binds a flavin adenine dinucleotide (FAD) complex. The FAD cofactor plays a key role in the oxidation of glucose and the final production of hydrogen peroxide (H_2_O_2_); FAD is reduced to FADH_2_ by glucose to produce hydrogen peroxide and D-glucono-*δ*-lactone, which is rapidly hydrolyzed to gluconic acid (Figure 1). The generated H_2_O_2_ can be electrochemically detected and further utilized for the quantification of glucose levels [7]. GOx can be immobilized on the nanomaterials via different methods such as physical adsorption, entrapment, cross-linking, and covalent binding. The immobilization method plays a critical role in the fabrication of the biosensor, as GOx should be capable of preserving its catalytic activity during the use of the biosensor [8,9]. Furthermore, the immobilization support should not interfere with the enzyme and should demonstrate faster response time acting as signal transducers [10,11].

Carbon nanotubes (CNTs) are cylindrical structures made of graphene sheets presenting different characteristics such as length, diameter, and number of layers, and they are categorized into single-walled carbon nanotubes (SWCNTs) and multi-walled carbon nanotubes (MWCNTs) [13,14]. CNTs are widely employed in the development of electrochemical biosensors, especially in glucose biosensing, as an effective immobilization matrix for GOx [13,15,16]; they facilitate the electron-transfer processes involved in the measurements and they endow improved detection capabilities due to their unique properties, including high strength, high electrical conductivity, advanced electrocatalytic properties, and high surface area-to-volume ratio [17,18]. Graphene, on the other hand, is a two-dimensional carbon nanomaterial whose atoms form a hexagonal crystal lattice [19,20,21]. Its large surface area provides a more extensive interface for enzyme immobilization, leading to higher sensitivity and lower detection limits [22,23]. Additionally, the exclusive electronic properties of graphene allow for efficient charge transfer between the electrode surface and the immobilized enzyme, resulting in improved biosensing performance [24,25]. Lastly, the combination of the two aforementioned carbon nanomaterials towards the development of hybrid composites for the design of glucose biosensors has also been reported. These hybrid structures are found to be promising supports for the immobilization of GOx, as they combine the unique properties of both parental nanomaterials [26,27].

However, both materials are generally considered hydrophobic, and as a result, their handling in aqueous solution recommended for enzymes, and other biomolecules, is difficult, whereas their low biocompatibility hinders their use in in vivo applications [28]. Bio-graphene (bG), a green-synthesized material, could be a promising candidate for the design and development of advanced electrochemical biosensors. bG is produced by liquid exfoliation using biomolecules, such as proteins, to both detach and stabilize the graphitic sheets [29,30]. bG offers great advantages due to its biocompatibility and the functional groups that are attached to its surface, combined with the special characteristics of graphene, rendering it an appropriate immobilization matrix for biosensing [29,31,32].

This study aimed to develop two monοenzymatic glucose biosensors, where MWCNTs and bG decorate the electrode surface and compose the support matrix for non-covalent immobilization of GOx. Before the development of the biosensors, a study on the effect of the two nanomaterials on the structural and biocatalytic characteristics of GOx was conducted, to deepen our understanding of activity–structure correlation. Ultraviolet-visible (UV-Vis), fluorescence, and circular dichroism (CD) spectroscopies were applied to evaluate changes arising from the presence of nanomaterials both in the overall structure of GOx, as well as in the FAD moiety of the enzyme, which is responsible for its oxidation activity. In the following step, the effect of MWCNTs and bG on the activity and the Michaelis–Menten apparent kinetic constants of GOx were determined and correlated to the structural changes. Finally, the calibration features of two electrochemical glucose biosensors, based on graphite screen-printed electrodes (SPEs) modified with an electropolymerized film of Prussian blue (PB), a very effective electron-transfer mediator for the electro reduction in the enzymatical produced H_2_O_2_ [33], and immobilized GOx in chitosan gels containing either bG or MWCNTs were compared and discussed.

## 2. Materials and Methods

### 2.1. Materials

Bovine serum albumin (BSA, 98% Fraction V), graphite powder (<20 μm, synthetic), glucose oxidase from *Aspergillus niger* (GOx, 215 U mg^−1^), horseradish peroxidase (HRP, 173 U mg^−1^), 2,2′-azino-bis(3-ethylbenzothiazoline-6-sulfonic acid) diammonium salt (ABTS), multi-walled carbon nanotubes (MWCNTs) short, O.D. × I.D. 40–70 nm × 5–40 nm × 0.5–2 μm, iron(III) chloride, chitosan (low molecular weight), and D(+)-glucose were purchased from Sigma-Aldrich (St. Louis, MO, USA). All the buffers and solutions were prepared by double-distilled water (ddH_2_O).

### 2.2. Synthesis of Bio-Graphene (bG)

Liquid exfoliation of graphite to produce bG was based on our previous work [29], with slight modifications. Briefly, 100 mg of graphite was added to 20 mL of ddH_2_O. The suspension was ultra-sonicated for 1 h (200 W, 10 kHz, pulse 50%), and the graphite flakes were partially exfoliated. In the next step, 100 mg of BSA was dissolved in 5 mL of ddH_2_O and then the protein solution was transferred to the graphite suspension. The graphite–protein mixture was stirred for 1 h at 25 °C. The unexfoliated graphite was separated by centrifugation at 2500 rpm for 10 min and the supernatant was collected carefully to acquire the lightest graphitic flakes. In the following step, the supernatant was subjected to three more centrifugations at 16,000 rpm for 60 min to remove the excess of BSA. After each centrifugation, the supernatant was disposed and the pellet, containing bG, was dispersed in ddH_2_O. Finally, 1 mL aliquot of the redispersed pellet was freeze-dried. The concentration of bG was calculated using Equation (1):

(1) $$bG\;concentration\;(mg/mL)=\frac{mg\;of\;freeze-dried\;powder}{mL\;of\;solution},$$

### 2.3. Determination of GOx Activity

GOx activity is determined by the coupling reaction of GOx with HRP; GOx catalyzes the conversion of D-glucose to D-glucono-*δ*-lactone and hydrogen peroxide, and in the following step, HRP oxidizes ABTS to ABTS^+^. Briefly, 200 mM of a glucose aqueous solution, 2.5 mM ABTS, 1 μg mL^−1^ of HRP, and 0.5 μg mL^−1^ of GOx were added in phosphate buffer (0.05 M, pH 7.0). Various volumes of aqueous dispersions of MWCNTs and bG were added to the reaction mixture to obtain final concentrations of nanomaterials in the range of 5–25 μg mL^−1^. The reaction was followed for 5 min by measuring the increase in absorbance of oxidized ABTS at 405 nm, using a UV/Vis spectrophotometer (Shimadzu, Tokyo, Japan). Enzymatic activity was expressed as the amount (μmoL) of ABTS transformed per minute.

The apparent kinetic constants (*V*_max_, *K*_M_,) of GOx in the presence or absence of bG and MWCNTs (25 μg mL^−1^) were calculated by measuring the initial reaction rates at 30 °C using different concentrations of glucose (0.25–25 mM).

### 2.4. Spectroscopic Characterization of GOx

UV–Vis spectra of GOx (500 μg mL^−1^) were recorded in phosphate buffer (0.05 M, pH 7.0) on a UV/Vis spectrophotometer (Shimadzu, Tokyo, Japan) in the presence of MWCNTs and bG (5–25 μg mL^−1^). The spectra were recorded in the range of 200–800 nm at room temperature. For each sample, a baseline was recorded, whereas two full wavelength scans were averaged for each sample.

Fluorescence measurements were recorded on a luminescence spectrofluorometer Jasco-8300 (Tokyo, Japan), using a 1 cm path-length quartz cuvette. A nominal bandpass of 5 nm was used for both the excitation and emission rays. The fluorescence emission spectra of GOx were recorded from 300 to 400 nm after excitation at 280 nm, associated with the tryptophan residue, as well as from 450 to 650 nm upon excitation at 373 nm, associated with the FAD moiety. In the first case, GOx was added at a concentration of 10 μg mL^−1^, whereas in the second one, GOx concentration was adjusted to 1 mg mL^−1^. In both cases, GOx emission spectra were recorded in the presence of MWCNTs and bG (5–25 μg mL^−1^). All emission measurements were taken in phosphate buffer (0.05 M, pH 7.0) at 25 °C. For every scanned sample, a baseline was recorded and subtracted from the sample spectrum. Each spectrum was recorded twice.

Circular dichroism (CD) measurements were performed at a Jasco J-1500 Circular Dichroism Spectrometer (Tokyo, Japan). The spectropolarimeter was equipped with a Peltier temperature control system. The far-UV CD spectra (200–260 nm) of GOx were recorded by the addition of GOx (25 μg mL^−1^) and MWCNTs or bG (at concentrations of 5 and 25 μg mL^−1^) to the phosphate buffer (0.01 M, pH 7.0). All spectra were obtained in a 1 cm quartz cuvette, with a 2 nm bandwidth and a scan rate of 50 nm min^−1^. For every sample, a baseline was recorded and subtracted from the protein spectrum, whereas at least two scans were averaged for each sample. The secondary structure content of GOx was estimated by implementing the K2D algorithm on the DichroWeb server [34].

### 2.5. Electrochemical Glucose Biosensors

Electrochemical measurements were conducted with a PGSTAT12/FRAII electrochemical analyzer (Metrohm Autolab, Utrecht, The Netherlands) using screen-printed three-electrode electrochemical cells (SPECs) connected to the electrochemical analyzer with a cable three-pin connector (Metrohm DropSens, Oviedo, Spain). Cyclic voltammetry (CV) experiments were conducted in 0.1 M phosphate buffer containing 0.1 M KCl, pH 6.0 at a scan rate of 50 mVs^−1^. Electrochemical impedance spectroscopy (EIS) measurements on SPECs modified with 5 μL of 2 + 1 *v/v* blend of 3 mg mL^−1^ bG in ddH_2_O, or 3 mg mL^−1^ MWCNTs in ddH_2_O with 1% *w/v* chitosan in 2% *v/v* acetic acid were recorded in 0.1 M phosphate buffer, pH 7.0 in 0.1 M KCl at 20 kHz using an excitation amplitude of 10 mV superimposed on DC potentials from −0.2 to 0.2 V (steps of 50 mV).

**Figure 2:**
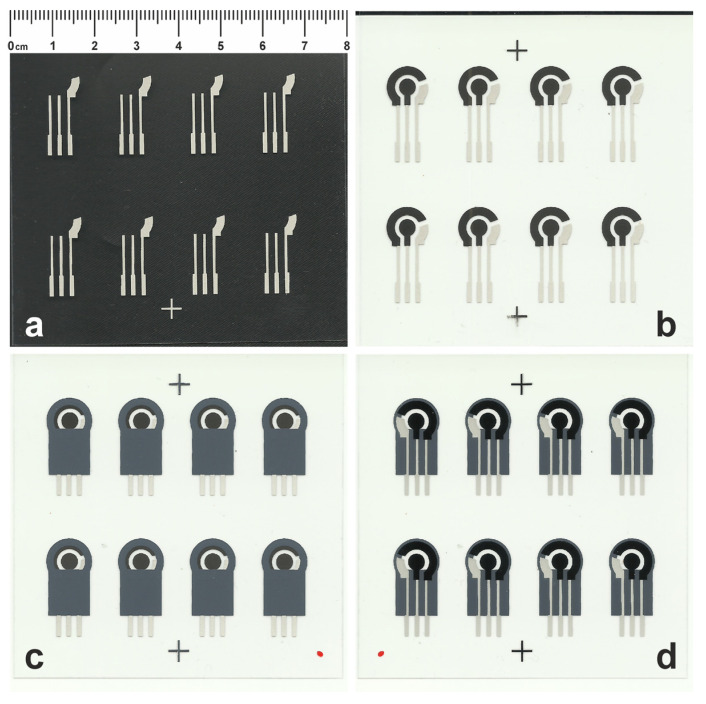
Photos showing the stepwise fabrication of SPECs after the (**a**) silver, (**b**) graphite, and (**c**) dielectric layers onto the polyester substrate. Photos (**c**,**d**) show the front and rear sides of the final electrochemical cells. Photo (**a**) was taken on a black background for clarity.

SPECs were fabricated in arrays of eight cells with a semi-automatic screen printer (E2, EKRA, Bönnigheim, Germany) onto a 175 μm thick polyester sheet (CUS7, Mac Dermid, Waterbury, CT, USA) using polyester screens (SEFAR PET 1500) and a 75-durometer polyurethane squeegee (Sefar AG, Heiden, Switzerland). SPECs consist of three layers printed in the following order: (a) a layer made of silver ink (Loctite EDAG 418SS E&C, Henkel, Düsseldorf, Germany), printed through a 90/230-48 PW screen, that acts as the conductive track for the working electrode (WE), the counter electrode (CE), and as a (Ag) pseudo-reference electrode (RE); (b) a layer made of graphite ink (Loctite EDAG 407A, Henkel), printed through a 77/195-48 PW screen, that serves as the CE and WE (4 mm diameter), and (c) a protective layer made of a dielectric ink (D2070423P5, Sun Chemical, Parsippany-Troy Hills, NJ, USA), printed through a 90/230-48 PW screen. After printing, the graphite ink was dried in a box oven (90 °C for 60 min), whereas the silver and the dielectric inks were dried in a dual-heater infrared conveyor dryer (Little Red X2, Vastex, Bethlehem, PA, USA) at 100 °C for 10 min. The stepwise fabrication of the SPECs is illustrated in Figure 2.

The modification of the WE surface was conducted by following a previously published protocol [15,33]. The graphite surface was electrochemically cleaned by applying 40 CV scans from −0.6 V to 1.6 V at 100 mVs^−1^ in 0.1 M phosphate buffer saline (PBS), pH 6.0. SPECs were rinsed thoroughly with ddH_2_O and dried with nitrogen. Then, 100 μL of 0.1 Μ HCl containing 5 mM FeCl_3_, 5 mM K_3_[Fe(CN)_6_], and 0.1 M KCl was pipetted on SPECs surface and an electropolymerized film of PB was developed onto the WE surface by applying 0.4 V for 60 s. PB/SPECs were rinsed thoroughly with ddH_2_O and an “activation” step was followed by applying 10 CV scans from −0.05 V to 0.35 V at 50 mVs^−1^ in 0.1 M HCl containing 0.1 M KCl. Then, PB/SPECs were rinsed thoroughly with ddH_2_O and dried with nitrogen. PB/SPECs was further modified by drop-casting on the WE surface a 5 μL of 2 + 1 *v/v* blend of 0.5 mg mL^−1^ GOx in 3 mg mL^−1^ bG in ddH_2_O, or 0.5 mg mL^−1^ GOx in 3 mg mL^−1^ MWCNTs in ddH_2_O with 1% *w/v* chitosan in 2% *v/v* acetic acid. Electrochemical biosensing of glucose was conducted by performing chronoamperometric measurements at −0.1 V for 30 s by applying 100 μL of glucose standards in 0.1 M phosphate buffer containing 0.1 M KCl, pH 6.0. Calibration curves were constructed by plotting the current value at 30 s with respect to the concentration of glucose.

## 3. Results and Discussion

### 3.1. Structural Characterization of GOx

The study of the intrinsic spectroscopic characteristics of an enzyme is a very prominent tool for investigating and comprehending the structural/conformational changes occurring during an enzymatic reaction, as the function of an enzyme strongly depends on its secondary and tertiary structure. Information regarding the tertiary structure, and more precisely the microenvironment around the aromatic amino acids of an enzyme, can be obtained by UV-Vis and fluorescence spectroscopy, whereas CD offers the advantage of determining the secondary structure of an enzyme. In this work, the effect of MWCNTs and bG on the spectroscopic characteristics of GOx was investigated. More specifically, UV-Vis and fluorescence spectroscopy were applied to study changes in the environment around the aromatic amino acids of GOx. These studies were extended to spectra recording in the FAD region. FAD is found buried in the active site of GOx and acts as the initial electron acceptor during an oxidizing reaction, thus changes around FAD moiety are of crucial importance. In the next step, an overall structural analysis was obtained via CD and fluorescence measurements to finally correlate structural-functional interactions.

#### 3.1.1. UV-Vis Spectroscopic Studies

**Figure 3:**
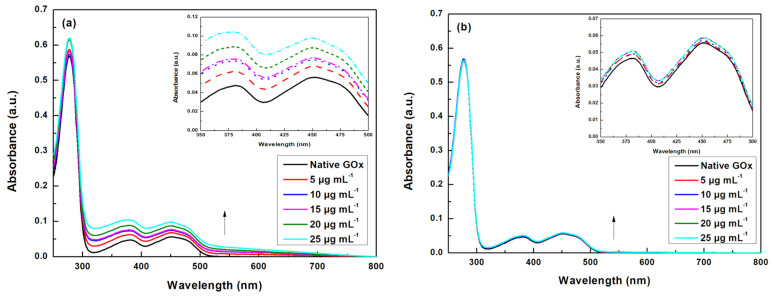
UV-Vis absorption spectra of GOx in the presence of different concentrations of (**a**) MWCNTs and (**b**) bG.

UV-Vis absorption spectroscopy was applied to investigate the possible modifications to the microenvironment around both the aromatic amino acids and the FAD moiety of GOx, due to the presence of the nanomaterials (Figure 3). The spectrum of GOx in the absence of nanomaterials exhibits a maximum absorbance value of 278 nm, corresponding to the aromatic amino acids present on the enzyme molecule. Moreover, the absorbance bands appearing at 382 and 452 nm are indicative of the presence of the FAD prosthetic group, with the band at 452 nm being slightly stronger than the one at 382 nm [35]. When MWCNTs are added to the GOx solution, the intensity of the band at 278 nm is slightly increased, insinuating that the MWCNTs affect the tertiary structure of GOx, by exposing the aromatic amino acids. The most pronounced changes are observed in the FAD absorption region; the intensity of the absorption bands at 382 and 452 nm increases with the addition of higher concentrations of the nanomaterial. This observation could be associated with changes in the FAD binding site, and more specifically exposure of the FAD moiety due to the unfolding of the enzyme in the presence of MWCNTs. A similar result was reported when graphene oxide was added to a free GOx solution, leading to a concentration-dependent increase in the absorption bands of the FAD moiety [36]. In the case of bG, the UV-Vis spectra of GOx do not present significant changes, even at high concentrations of bG, showing that the enzyme maintains its natural form.

#### 3.1.2. Fluorescence Studies

**Figure 4:**
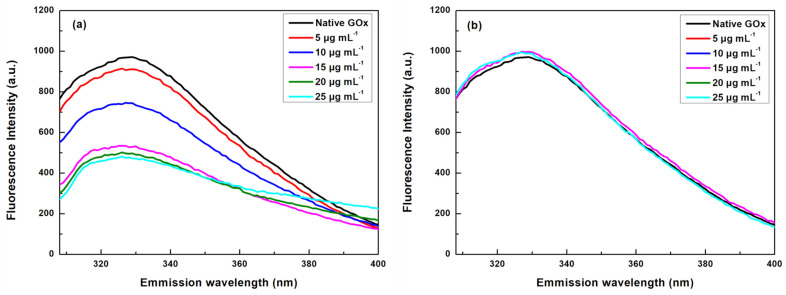
Fluorescence emission spectra of GOx in the presence of different concentrations of (**a**) MWCNTs and (**b**) bG, in the Trp region after excitation at 280 nm.

The conformational changes around the aromatic amino acids and FAD moiety were also studied by fluorescence spectroscopy. Most of the proteins consist of fluorescent amino acid residues, namely tryptophan (Trp), tyrosine (Tyr), and phenylalanine (Phe). The Trp residues can be selectively excited to provide more precise structural information in proteins due to their sensitivity to environmental changes. Each subunit of a GOx molecule contains ten Trp residues; four of them are found in the FAD moiety [37]. The fluorescence emission spectra of GOx in the presence of increasing concentrations of MWCNTs and bG were recorded, and the results are presented in Figure 4. The results show that the presence of MWCNTs quenches the Trp fluorescence to a high extent (Figure 4a). In addition, a blue shift from 329 (native form) to 326 nm is observed when the concentration of the nanomaterial increases, suggesting that structural changes take place at the tertiary arrangement of the polypeptide chain, revealing alterations in the polarity in the vicinity of Trp residues [38]. Typically, a blue shift demonstrates the transmission of Trp residues to an environment of higher hydrophobicity, proving that MWCNTs increase the hydrophobicity around the Trp residues [36]. Contrary to MWCNTs, bG does not affect the emission spectra of GOx, indicating that the enzyme preserves its tertiary structure (Figure 4b).

**Figure 5:**
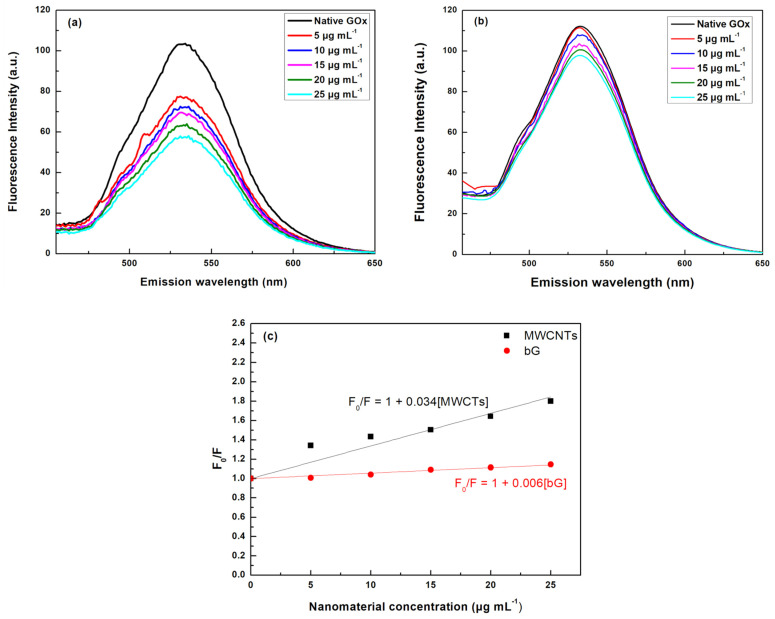
Fluorescence emission spectra of GOx in the presence of different concentrations of (**a**) MWCNTs, and (**b**) bG, in the FAD moiety region after excitation at 373 nm, and (**c**) Stern–Volmer curves in the presence of MWCNTs and bG.

The conformation changes around the FAD prosthetic group were also studied by fluorescence measurements, as FAD is known to be very sensitive to alterations in its microenvironment [39]. In the native form of GOx, the FAD moiety exhibits an emission maximum at approximately 534 nm upon excitation at 373 nm; this fluorescence band is associated with FAD in its oxidized form [40]. In the presence of MWCNTs, significant alterations in the FAD emission spectra are observed (Figure 5a). MWCNTs present a concentration-dependent quenching effect. This fluorescence quenching, due to the increased concentrations of MWCNTs, could result from alterations in the microenvironment around the FAD prosthetic group. On the contrary, the presence of bG does not affect the emission spectra of FAD (Figure 5b), indicating that the surface characteristics of the nanomaterials play a critical role in the fluorescence characteristics of GOx. MWCNTs due to their hydrophobic nature, create a more hydrophobic environment, whereas bG is mostly hydrophilic, owing to the presence of albumin, thus offering a more hydrophilic environment around the FAD moiety [32].

The Stern–Volmer (K_sv_) constants, which describe the quenching rate, were also determined, and they are presented in Figure 5c. The Stern–Volmer equation relates the decrease in fluorescence intensity to the concentration of a collisional quencher, in our case the nanomaterials, and is described by Equation (2):

(2) $$F_{0}/F=1+K_{sv}[Q]$$

where, F_0_ and F are the fluorescence intensity in the absence and in the presence of the quenching agent, respectively, [Q] is the concentration of the quenching agent, and K_sv_ is the Stern–Volmer quenching constant. The K_sv_ of GOx in the presence of MWCNTs and bG were calculated at 0.034 and 0.006 μg mL^−1^, respectively. The higher K_sv_ in the case of MWCNTs suggests that the space between the fluorophore and the quencher decreases, and thus the aromatic amino acids around the FAD moiety of GOx are exposed and are easily accessible to MWCNTs, resulting in stronger interactions [41,42,43]. These results agree with the UV-Vis measurements, discussed above.

#### 3.1.3. Circular Dichroism Spectroscopy

**Figure 6:**
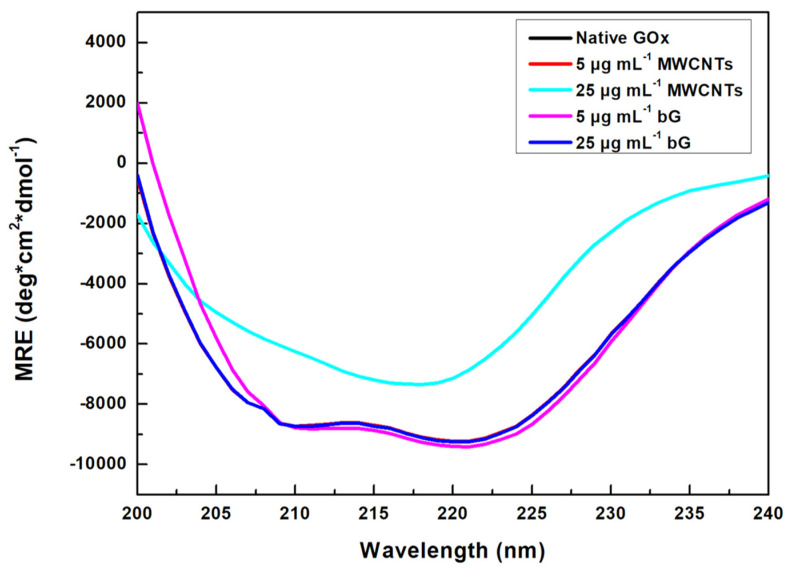
Far-UV CD spectra of GOx in the presence of MWCNTs and bG.

CD spectroscopy was employed to study the structural changes in the GOx molecule in the presence of the two nanomaterials (MWCNTs and bG) (Figure 6). The CD spectrum in the far-UV region of the native GOx depicts two negative peaks at around 208 nm and 221 nm, characteristic of an *α*-helix structure [44,45]. The far-UV spectrum of GOx remains unchanged in the presence of 5 μg mL^−1^ MWCNTs; however, when the concentration of the nanomaterial increases to 25 μg mL^−1^, significant alterations are observed. More specifically, the band at 208 nm almost disappears, whereas the appearance of a band at 218 nm is associated with a *β*-sheet conformation, indicating severe changes in the secondary structure of GOx. On the contrary, the presence of bG does not affect the far-UV CD spectra of GOx, demonstrating the zero effect of bG in GOx secondary structure.

The CD spectra of GOx in the absence and the presence of the two nanomaterials were further analyzed by DichroWeb, and the results are compiled in Table 1. The native form of GOx consists of 32% *α*-helices, 12% *β*-sheets, and 56% random coil. The presence of 5 μg mL^−1^ MWCNTs does not affect the secondary structure of the enzyme, as already commented (Figure 6), whereas, at a nanomaterial concentration of 25 μg mL^−1^, GOx undergoes severe conformational changes; the *α*-helical structure decreases to 19%, whereas the *β*-sheet content increases to 29%. This transition of *α*-helix to *β*-sheet implies that GOx adopted a looser conformation in the presence of high concentrations of MWCNTs [46]. The results of CD are following the results obtained by UV-Vis and fluorescence measurements, proving that high concentrations of MWCNTs affect the structural characteristics of GOx. In the case of bG, the secondary structure of GOx is not significantly altered, indicating that bG offers a protective effect on the secondary structure of the enzyme.

### 3.2. Biocatalytic Activity of GOx

**Figure 7:**
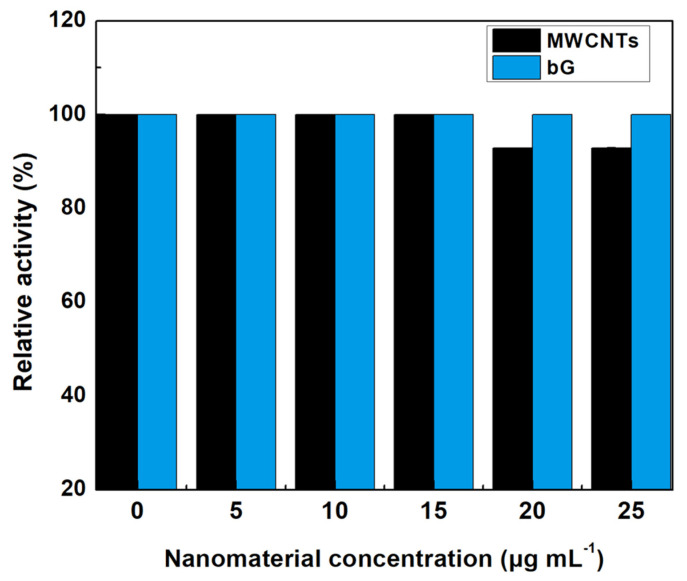
Relative activity of free GOx in the presence of different concentrations of MWCNTs and bG. One hundred percent indicates the activity of native GOx in the absence of nanomaterials (the standard deviation was <0.2% in all cases, and thus error bars are not possible to be seen).

As already discussed above, the activity of an enzyme strongly depends on its structure/conformation. After demonstrating the effect of MWCNTs and bG on the structure of GOx, we proceeded to investigate the impact of the changes on the oxidizing activity of the enzyme. For activity detection, an HRP-conjugated reaction was used. The activity of GOx was determined in the presence of increasing concentrations of MWCNTs and bG (Figure 7). As can be seen, the presence of bG does not affect the oxidation activity of GOx, whereas MWCΝTs decrease the enzymatic activity when added in high concentrations (20 and 25 μg mL^−1^). These results agree with the spectroscopic data that reflect the influence of MWCNTs on the structure of GOx. More specifically, the transition from an *α*-helical structure to a *β*-sheet conformation affects the catalytic performance of GOx. Being the fundamental protein backbone, *α*-helix is more compact than the other secondary forms; thus, it is anticipated to shield the protein from external factors [47]. The α-helical loss is usually associated with activity loss [48]. The loss of this conformation in the presence of MWCNTs promotes the formation of the loose *β*-sheet which induces a significant conformational change in the enzyme affecting its catalytic activity [36]. On the contrary, bG protects GOx from misfolding and thus preserves its oxidation activity. This protective effect of bG may arise from its surface characteristics; BSA-decorated graphene flakes, due to the presence of the protein, offer a more compatible environment to the enzyme [49]. It has been previously shown, that bG can be effectively used as an immobilization carrier for GOx, by preserving its catalytic characteristics [29].

To further examine the effect of the two nanomaterials on the catalytic performance of GOx, the apparent kinetic constants (*V*_max_ and *K*_M_) of the enzyme were determined in the presence of 25 μg mL^−1^ bG and MWCNTs (Table 2). In the case of MWCNTs, both kinetic constants of GOx decrease by 10%. The decrease in the *V*_max_ value signifies a restricted oxidation rate, which could be connected to the structural changes observed in the presence of the nanomaterial. UV-Vis and fluorescence spectra revealed significant alterations around the FAD binding site in the presence of high concentrations of MWCNTs. As FAD is responsible for the oxidative activity of GOx, the modifications near the binding site of the prosthetic group have a substantial impact on the activity of the enzyme, which is mirrored by the decline of the *V*_max_ value. Moreover, the increase in the *K*_M_ value is associated with decreased affinity of the enzyme towards its substrate [29]. The conformational changes in the secondary structure of GOx may lead to changes in the binding efficacy towards glucose, supporting the decrease in the catalytic activity. As far as it concerns the case of bG, the *V*_max_ of GOx only slightly decreases, whereas a 10% decrease in the *K*_M_ value is also recorded, hinting at a slight increase in the affinity of the enzyme towards the substrate. This result follows the results that were previously discussed, reinforcing the fact that bG only positively affects GOx compared to MWCNTs.

### 3.3. Performance of Electrochemical Glucose Biosensors

**Figure 8:**
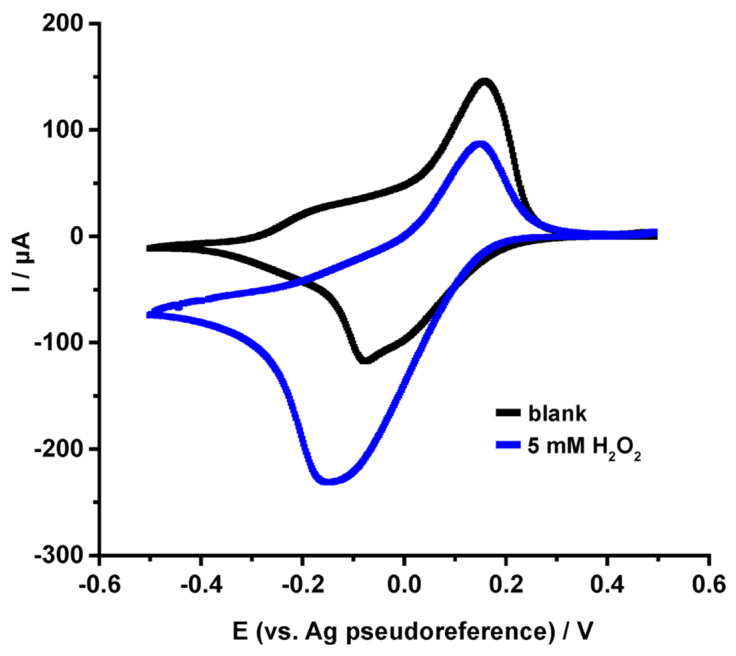
Cyclic voltammograms of PB/SPEC in the (black) absence and (blue) presence of 5 mM H_2_O_2_ in 0.1 M phosphate buffer containing 0.1 M KCl, pH 6.0. Scan rate, 50 mVs^−1^.

The cyclic voltammogram illustrated in Figure 8, exhibits a pair of well-defined oxidation/reduction peaks, at a formal redox potential (E^o’^ = 131 mV vs. Ag pseudo-reference electrode). These peaks are attributed to the redox transition between the PB and Prussian white (PW, the reduced form of PB) and confirm the effective formation of the PB electropolymerized film on the surface of WE [50]. The peak potential separation value was calculated ΔΕ_p_ = 263 mV and the deviation from the ideal value of 0 mV expected from an immobilized redox molecule can be attributed both to the interactions between the immobilized molecules and with the electrode surface as well as to the diffusion of K^+^ ions involved in the redox reaction to compensate the transfer of the electrons, according to Equation (3) [51]:

KFe^(III)^[Fe^(II)^(CN)_6_] (PB) + K^+^ + e^−^ ⇌ K_2_Fe^(II)^[Fe^(II)^(CN)_6_] (PW) (3)

The excellent electrocatalytic properties of PB/SPE towards the electro-reduction in H_2_O_2_ are demonstrated by the cyclic voltammograms illustrated in Figure 8. In the presence of H_2_O_2_, during the cathodic scan, a substantial increase in the current, very close to the potential of the PW, can be explained considering the diffusion of H_2_O_2_ on the electrode surface and the chemical oxidation of PW to PB that is then electrochemically reduced to PW during the cathodic sweep of the potential.

**Figure 9:**
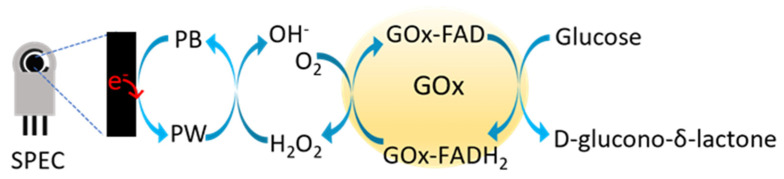
Schematic representation of reactions involved for the determination of glucose with the GOx-modified PB/SPEC.

The electrochemical sensing properties of PB/SPECs, after their modification with a 2 + 1 *v/v* blend of 0.5 mg mL^−1^ GOx in 3 mg mL^−1^ bG (bG-GOx), or 0.5 mg mL^−1^ GOx in 3 mg mL^−1^ MWCNTs in ddH_2_O (MWCNT-GOx), with 1% *w/v* chitosan in 2% *v/v* acetic acid were also examined. The schematic representation of the reactions involved in the determination of glucose with the GOx-modified PB/SPEC is illustrated in Figure 9.

**Figure 10:**
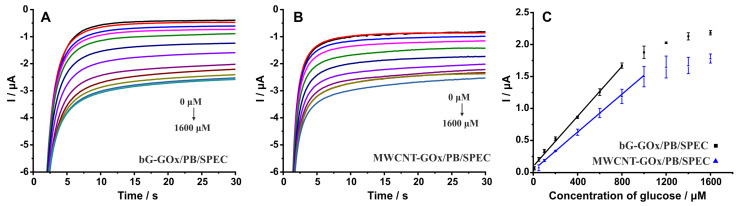
Representative chronoamperometric response of (**A**) bG-GOx/PB/SPEC, (**B**) MWCNT-GOx/PB/SPEC at various concentrations of glucose (0, 10, 50, 100, 200, 400, 600, 800, 1000, 1200, 1400, and 1600 μM), and (**C**) the respective calibration plots based on the mean values of the current at 30 s obtained by four different biosensors in each case. Error bars represent the standard deviation of the measurements. Chronoamperometric plots were conducted at −0.1 V in 0.1 M phosphate buffer, pH 6.0 containing 0.1 M KCl.

The chronoamperometric scans and the respective calibration plots illustrated in Figure 10 demonstrate that bG-GOx/PB/SPECs exhibit a linear response over the concentration range 10–800 μΜ glucose with the data fitted the equation I (μA) = 0.10 + 0.00195 (C_glu_/μM), R^2^ = 0.9935, whereas MWCNT-GOx/PB/SPECs exhibit a linear response over the concentration range 50–1000 μΜ glucose with the data fitted the equation I (μA) = 0.04 + 0.00148 (C_glu_/μM), R^2^ = 0.9970. Based on the slope values in each case, the sensitivity of the measurements (μA/μΜ/cm^2^) at bG-GOx/PB/SPECs is 1.33-fold higher compared with that obtained at MWCNT-GOx/PB/SPECs.

In addition, the electrochemical properties of the working electrode/electrolyte interface were further examined by performing EIS experiments at electrodes modified with a film of bG/chitosan and MWCNT/chitosan to evaluate the effect of each material on the conductivity of the respective modifying polymer layers. Measurements of the real impedance component were conducted in the absence of the electropolymerized PB film and any redox probe in the electrolyte to avoid any faradaic response, and at 20 kHz to drastically suppress the contribution of any capacitive currents (that is, the capacitance of the double layer and the film capacitance) to the overall impedance. In this regard, the recorded impedance can be attributed to the total ohmic resistance due to the resistance of the electrolyte, the connection cables, the working electrode itself, and the modifying layer. Considering that the three first contributions remain constant, the measured impedance is largely attributed to the modifying film resistance [52]. For three different electrodes, the means and the standard deviation of the measurements of real impedance values extracted from the plot Z_real_ = f(V) at the voltage −0.1 V, were found to be 1.21 ± 0.03 (n = 3) Ohm for bG/chitosan/SPEC and 1.24 ± 0.08 Ohm for MWCNT/chitosan/SPEC indicating that bG-based chitosan films are slightly more conductive. Standard deviation values in each case also demonstrate that hydrophilic bG results in more reproducible modifying coatings, and consequently, in more reproducible biosensors, in agreement with the measurements illustrated in Figure 10C.

## 4. Conclusions

This work aimed at the development of glucose biosensors fabricated with a widely applied nanomaterial, MWCNTs, as well as with a novel graphene derivative, bio-graphene (bG). Initially, the two nanomaterials were evaluated for their effect on the structural/conformational characteristics of GOx. UV-Vis, fluorescence, and CD results demonstrated that the presence of bG does not significantly affect the structure of GOx, whereas pronounced changes were detected in the presence of MWCΝTs. Both the secondary structure and FAD binding site are altered upon interactions between GOx and MWCNTs, leading to an observed decrease in the oxidation activity and *V*_max_ of the enzyme and an increase in its *K*_M_ value. The beneficial properties of bG over MWCNTs on GOx activity were further supported by electrochemical data at two glucose biosensors based on GOx entrapped in chitosan gel in the presence of bG or MWCNTs. bG-based biosensors gave a 1.33-fold increased sensitivity for the determination of glucose as well as an improved reproducibility reflecting the adverse effect of MWCNTs on the structure and activity of GOx and the increased hydrophilicity of bG-based aqueous suspensions, which ensure an even distribution of the respective modifying films onto the electrode surface, respectively.

## Figures and Tables

**Table 1:** Secondary structure analysis of GOx in the presence of MWCTNs and bG.

Sample	*α*-Helix (%)	*β*-Sheet (%)	Random Coil (%)
Native GOx	32	12	56
GOx + 5 μg mL^−1^ MWCNTs	31	10	59
GOx + 25 μg mL^−1^ MWCNTs	19	29	52
GOx + 5 μg mL^−1^ bG	32	12	56
GOx + 25 μg mL^−1^ bG	31	10	59

**Table 2:** Apparent kinetic constants of GOx in the presence of bG and MWCNTs.

Sample	*V*_max_ (μM min^−1^)	*K*_M_ (mM)
Native GOx	7.00 ± 0.51	1.70 ± 0.45
GOx + 25 μg mL^−1^ MWCNTs	6.30 ± 0.20	1.87 ± 0.20
GOx + 25 μg mL^−1^ bG	6.73 ± 0.26	1.52 ± 0.22

## Data Availability

Data are contained within the article.
